# Changes in serum zinc and copper concentrations in patients with cardiovascular disease following cardiac surgery

**DOI:** 10.14814/phy2.15483

**Published:** 2022-10-06

**Authors:** Zahra Moravvej, Vafa Baradaran Rahimi, Ali Azari, Amir Ali Rahsepar, Majid Ghayour‐Mobarhan, Maryam Salehi, Leila Bigdelu

**Affiliations:** ^1^ Division of Cardiovascular, Vascular Surgery Research Center Mashhad University of Medical Sciences Mashhad Iran; ^2^ Department of Ophthalmology, Hakim Hospital Neyshabur University of Medical Sciences Neyshabur Iran; ^3^ Department of Cardiovascular Diseases, Faculty of Medicine Mashhad University of Medical Sciences Mashhad Iran; ^4^ Division of Cardiology, Department of Medicine Johns Hopkins University School of Medicine Baltimore Maryland USA; ^5^ Metabolic Syndrome Research Center Mashhad University of Medical Sciences Mashhad Iran; ^6^ International UNESCO Center for Health‐Related Basic Sciences and Human Nutrition Mashhad University of Medical Sciences Mashhad Iran; ^7^ Department of Community Medicine School of Medicine, Mashhad University of Medical Sciences Mashhad Iran

**Keywords:** copper, coronary artery disease, trace element, valve replacement, zinc

## Abstract

The trace elements copper (Cu) and zinc (Zn) are essential for maintaining oxidative balance, and cardiac surgery is known to provoke an increase in oxidative stress. We investigated the variations in serum Zn and Cu concentrations before and after surgery in patients undergoing on‐ and off‐pump CABG and heart valve replacement. We performed a prospective study on patients undergoing on‐ or off‐pump CABG, or heart valve replacement surgery (48, 51, and 47 patients, respectively). Venous blood samples were obtained, and serum Cu and Zn concentrations were measured preoperatively, 24 h postoperatively, and the time of discharge. In addition, echocardiography was carried out on all patients before surgery and again on the day of discharge. We found the temporal changes in Cu, Zn, and Zn/Cu ratio were significantly different in all three groups of surgery (*p* < 0.05). In each group, Cu and Zn values and Zn/Cu ratio decreased at the 24‐h postoperative time and rose at the discharge time. There were no significant differences between surgery groups in the changes induced in Zn or Cu values (*p* > 0.05). In conclusion, the concentrations of Cu and Zn were markedly reduced after on‐ and off‐pump CABG and valve replacement surgery. This may suggest that supplementary Zn and Cu administration could be beneficial during open‐heart surgeries. However, more long‐term studies with more patients are needed to confirm this hypothesis.

## Graphical abstract

In conclusion, the concentrations of Cu and Zn were markedly reduced after on‐ and off‐pump CABG and valve replacement surgery. Copper concentrations were inversely associated with cardiopulmonary bypass duration, implying the need to evaluate the precise role of CBP in inducing oxidative stress.

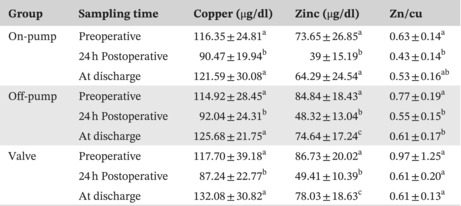

## 1 INTRODUCTION

Cardiovascular disease (CVD) is the leading cause of death and disability worldwide (Bigdelu et al., 2018). Myocardial revascularizations aiming to treat CVD can be accomplished percutaneously or surgically, known as coronary artery bypass grafting (CABG) (Gholoobi et al., 2021). The standard surgical intervention for coronary revascularization includes cardiopulmonary bypass (CPB) and cardioplegia‐induced cardiac arrest, defined as the “on‐pump” technique. Off‐pump CABG is considered the newer method and performed with the heart beating and without CPB (Shekar, 2006). Although valve replacement surgery and CABG with the use of CPB have similar intraoperative procedures, the patients in these two categories have different pathologies, which may alter their responses to similar injuries (Bigdelu et al., 2022; Rahsepar et al., 2013).

The study of trace element metabolism is greatly valued as it provides insights into understanding preventive, diagnostic, and therapeutic mechanisms. Much evidence has been produced indicating that manganese, iron, copper (Cu), zinc (Zn), and selenium are significant elements in the field of coronary heart disease and coronary artery heart bypass grafting research (Admans et al., 2006; Azari et al., 2015). The ability of Zn to retard oxidative processes has been recognized for many years. Zinc can reduce postischemic injury to various tissues and organs through a mechanism that might involve the antagonism of Cu reactivity (Baradaran Rahimi et al., 2019). The trace elements Cu and Zn are essential for maintaining oxidative balance (Yan & Zou, 2012). In cardiopulmonary bypass surgery, an exaggerated systemic inflammatory response syndrome (SIRS) and systemic oxidative stress appear to give rise to many post‐surgery complications (Askari et al., 2020). The present study aimed to evaluate Zn and Cu level variations among three groups of patients undergoing on‐pump and off‐pump CABG and valvular heart replacement surgery.

## 2 METHODS AND MATERIALS

The patients were candidates for open‐heart surgery and had proven coronary artery disease (CAD) or valvular heart disease (VHD). The inclusion criteria were patients over the age of 18 years with CAD undergoing CABG for the first time and also patients with VHD undergoing valvular surgery. Patients with the following conditions were excluded from the study: taking oral contraceptives or hormone replacement therapy, pregnancy, prior history of coronary angioplasty or CABG, chronic pulmonary obstructive disease, overt clinical features of infection, acute coronary syndrome within the previous 2 weeks, renal, hepatic or malignant diseases, and cases treated as emergencies. The indication for coronary revascularization and the type of surgery (off‐ or on‐pump CABG) were determined based on clinical judgment by the attending cardiac surgeon. Data were obtained from 51 patients in off‐pump CABG, 48 patients in on‐pump CABG, and 47 patients in the VHD group. According to guidelines, percutaneous coronary angiography was indicated for patients with VHD in order to rule out the presence of CAD. The angiography results showed that all patients with VHD were free from CAD and without any stenosis.

Demographic and intraoperative data were recorded, including the aortic cross‐clamp and cardio‐pulmonary bypass (CPB) times. In addition, venous blood samples were taken from each patient for analysis after a 12‐h fast, on the morning of the day of surgery (Preoperative time) and the first postoperative day at 24 h after surgery (24 h Postoperative), while the patient was in the intensive care unit (ICU). A third sample was taken at the time of discharge after a 12‐h fast. Blood samples were collected into plain Vacutainer® tubes and centrifuged at 10,000*g* for 15 min at 4°C. After separation, aliquots of serum were frozen at −80°C until analysis (Rahsepar et al., 2011). Serum Zn and Cu concentrations were, respectively, measured spectrophotometrically using commercial available Biorex BXC0462 and BXC0341 kits on an autoanalyzer (model BT3000, Biotechnica) at the Faculty of Medicine, Mashhad University of medical sciences. The values are expressed in μg/dl. The normal reference value for Zn is 72.6–127 μg/dl and 70–114 μg/dl in men and women, respectively. The normal reference value for Cu is 70–140 μg/dl and 80–155 μg/dl in men and women, respectively.

Echocardiographic examinations were performed on all patients before surgery and again on the day of discharge, using a VIVID 3 instrument (GE Vingmed Ultrasound). The left ventricular (LV) ejection fraction (EF), LV end‐systolic and end‐diastolic diameters and volumes, early (E) and late (A) mitral forward Doppler flow, early (E′) and late (A′) diastolic mitral annulus pulsed‐wave tissue Doppler, and end‐systolic and end‐diastolic volumes were measured. E/E′ was indicated as the pulmonary capillary wedge pressure.

The study protocol was approved by the ethics committee of Mashhad University of Medical Sciences, and written informed consent was obtained from each participant.

### 2.1 Statistical analyses

Statistical analyses were performed using SPSS for Windows, version 16 software. Normal distribution was tested by the Kolmogorov–Smirnov test. Descriptive data were expressed as mean ± SD for parameters with a normal distribution, as a median and interquartile range in the case of non‐normally distributed data, and as a percentage when required. The associations between baseline concentrations, patient characteristics, and echocardiographic parameters were examined using Pearson's correlation test. Also, the associations of CPB or aortic clamp time and Zn and Cu level variations before and after surgery were examined using Pearson's correlation test. Comparisons were made using one‐way ANOVA (for three groups) or the Student's *t*‐test (between two groups) for baseline Zn and Cu concentrations. The repeated measure ANOVA accounted for the correlated observations within the groups. We included as fixed effects the grouping factor treatment (on‐pump, off‐pump, and valve replacement surgery) and the within‐factor sampling time. Mean comparisons were performed using Bonferroni multiple comparison tests. A *p*‐value <0.05 was considered significant.

## 3 RESULTS

### 3.1 Demographic data

Overall, 48, 51, and 47 patients were evaluated in the on‐pump, off‐pump CABG, and valve replacement groups, respectively (Table 1). In the study groups, no hospital mortality, no neurological accidents, and incidences of myocardial infarction occurred. In the on‐pump and off‐pump CABG and valve replacement groups, 50% versus 50%, 60.8% versus 39.2%, and 46.8% versus 53.2% of the patients were men and women, respectively. Baseline and intraoperative characteristics are given in Table 1.

**TABLE 1:** Baseline and intraoperative characteristics of patients

Parameters	Patient group		
	On‐pump	Off‐pump	VHD
Patients (*n*)	48	51	47
Age (years)^a^	62.10 ± 9.80	58.18 ± 9.10	43.55 ± 13.95
Gender (male/female)	24/24	31/24	22/25
Diabetic (*n*, %)	17 (35.4)	19 (37.3)	2 (4.3)
Smoker (*n*, %)	14 (29.2)	19 (37.3)	10 (21.3)
Hypertension (*n*, %)	30 (62.5)	33 (64.7)	10 (21.3)
Dyslipidemia (*n*, %)	18 (37.5)	30 (58.8)	5 (10.6)
BMI (kg/m^2^)^a^	26.42 ± 4.83	27.11 ± 3.69	23.24 ± 4.51
SBP^b^	130 (115–137)	120 (110–130)	115 (110–130)
DBP^b^	80 (72.5–82.5)	80 (70–80)	70 (67.5–81)
Aortic cross‐clamp time (min)^b^	50 (45–64)	—	58 (36–93.25)
Cardiopulmonary bypass time (min)^b^	87 (68–113)	—	73 (54.25–109)
Number of grafts (*n*)^a^	3.06 ± 0.78	3.18 ± 1.03	—
Hospitalization (days)^b^	8 (7–9.25)	7 (7–8)	8 (7.75–11)
Coronary lesions (stenosis ≥70)			—
LAD	4 (8.3)	5 (9.8)	
LAD + RCA	5 (10.4)	4 (7.8)	
LAD + LCx	5 (10.4)	7 (13.7)	
LAD + RCA + LCx	34 (70.8)	34 (66.7)	
RCA + LCx	0	1 (2)	
Number of valve replacements	—	—	
One			35 (74.5)
Two			8 (17)
Three			4 (8.5)

*Note*: Other values in parentheses are percentages.

Abbreviations: BMI, Body mass index; DBP, Diastolic blood pressure; LAD, Left anterior descending artery; LCx, Left circumflex artery; RCA, Right coronary artery; SBP, Systolic blood pressure; VHD, Valvular heart disease.

^a^ Values are mean ± SD.

^b^ Values are median and interquartile range.

### 3.2 Associations with baseline Cu and Zn

CAD and VHD patients were grouped according to hypertension, diabetes, dyslipidemia, and smoking. Baseline Cu and Zn values were compared between groups (Table 2). In CAD patients, the results showed a higher baseline Zn value in non‐hypertensive cases (89.18 vs. 73.89 μg/dl; *p* = 0.002) and a lower baseline Zn value in non‐smoking cases (76.14 vs. 86.76 μg/dl; *p* = 0.046). In VHD patients, baseline Cu values were significantly lower in non‐dyslipidemic patients (114.62 vs. 168.67 μg/dl; *p* = 0.017) (Table 2).

**TABLE 2:** Baseline concentrations (mean ± SD) of copper and zinc in various groups of patients

Parameters	Group	CAD		VHD	
		Copper (μg/dl)	Zinc (μg/dl)	Copper (μg/dl)	Zinc (μg/dl)
Hypertension	No	129.29 ± 30.42	89.19 ± 21.98^a^	116.26 ± 39.66	87.11 ± 19.67
	Yes	123.32 ± 40.75	73.89 ± 21.59	127.75 ± 33.52	84.37 ± 22.85
Diabetic	No	119.71 ± 35.22	76.27 ± 20.69	116.37 ± 37.75	85.63 ± 19.79
	Yes	134.94 ± 39.41	84.30 ± 25.52	160.00 ± 39.59	106.50 ± 19.09
Dyslipidemia	No	127.39 ± 40.91	78.32 ± 23.35	114.62 ± 36.86^a^	85.22 ± 19.23
	Yes	123.26 ± 33.41	80.33 ± 22.46	168.67 ± 23.15	105.00 ± 25.98
Smoker	No	123.71 ± 29.03	76.14 ± 20.59^a^	113.03 ± 36.11	83.96 ± 19.62
	Yes	129.50 ± 52.70	86.76 ± 26.35	136.10 ± 42.69	95.30 ± 19.88

Abbreviations: CAD, Coronary artery disease; VHD, Valvular heart disease.

^a^ For each characteristic mean values are significantly different between two groups (independent *t*‐test at *p* < 0.05).

Associations between baseline Cu and Zn values and age, BMI, systolic, and diastolic blood pressures were investigated in patients with CAD. The results showed no correlation between the above factors and baseline Cu and Zn values (*p* > 0.05). In addition, the relationship between baseline Cu and Zn values and echocardiographic parameters was assessed in patients with CAD. There were no significant associations shown regarding baseline Cu and Zn values and echocardiographic findings, except that E/E′ had a significant negative association with baseline Zn values (*p* = 0.048, *r* = −0.248) (Table 3).

**TABLE 3:** Correlation between baseline copper and zinc values and different parameters

Parameters	CAD				VHD			
	Copper (μg/dl)		Zinc (μg/dl)		Copper (μg/dl)		Zinc (μg/dl)	
	*r*‐value^a^	*p*‐value	*r*‐value^a^	*p*‐value	*r*‐value^a^	*p*‐value	*r*‐value^a^	*p*‐value
Age	−0.085	0.433	−0.148	0.170	0.195	0.210	−0.261	0.091
BMI	0.135	0.217	0.049	0.656	0.101	0.529	0.159	0.321
DBP	0.062	0.576	−0.051	0.647	−0.164	0.307	0.016	0.921
SBP	−0.005	0.966	−0.139	0.208	−0.163	0.309	0.006	0.968
EF	−0.151	0.164	−0.130	0.233	0.252	0.103	0.088	0.574
ESV	0.146	0.265	0.102	0.439	−0.316	0.068	0.015	0.933
EDV	0.127	0.333	0.023	0.864	−0.293	0.092	0.123	0.489
LVDD	0.158	0.159	0.158	0.158	−0.272	0.120	−0.054	0.763
LVSD	0.133	0.235	0.152	0.175	−0.266	0.141	−0.385	0.030
E/E′	0.014	0.911	−0.248	0.048	−0.023	0.897	−0.118	0.506
EF_1‐2_	−0.095	0.412	−0.054	0.646	0.107	0.527	0.052	0.758
ESV_1‐2_	−0.027	0.875	0.026	0.877	0.022	0.918	0.149	0.486
EDV_1‐2_	−0.013	0.940	0.056	0.742	−0.080	0.709	0.180	0.401
LVDD_1‐2_	−0.040	0.781	−0.227	0.113	0.003	0.989	−0.062	0.772
LVSD_1‐2_	−0.036	0.802	−0.268	0.060	−0.189	0.387	−0.400	0.059
E/E′_1–2_	−0.159	0.301	−0.102	0.511	0.037	0.861	−0.036	0.866

Abbreviations: BMI, Body mass index; CAD, Coronary artery disease; DBP, Diastolic blood pressure; E′, Peak early diastolic myocardial velocity; E/E′, Pulmonary capillary wedge pressure; E, Peak early trans‐mitral flow velocity; EDV, End‐diastolic volume; EF, Ejection fraction; ESV, End‐systolic volume; LVDD, Left ventricular diastolic diameter; LVSD, Left ventricular systolic diameter; SBP, Systolic blood pressure; VHD, Valvular heart disease.

a Correlations were assessed using Pearson’s correlation test.

Associations between baseline Cu and Zn values and age, BMI, systolic and diastolic blood pressures were investigated in patients with VHD. The results showed no correlation between the above factors and baseline Cu and Zn values (*p* > 0.05). In addition, no significant associations were demonstrated between baseline Cu and Zn values and echocardiographic findings, except for LV systolic diameter, which was negatively associated with baseline Zn levels (*p* = 0.030, *r* = −0.385, Table 3). Furthermore, the changes in echocardiographic parameters and baseline Cu and Zn values were assessed, and values were not related to changes in echocardiographic findings (Table 3). Moreover, diastolic function was not associated with baseline Cu or Zn values.

### 3.3 Copper values

There was no significant difference in baseline Cu values among the patients' off‐ and on‐pump CABG and valve replacement groups (*p* = 0.183). Analysis by repeated measure ANOVA showed that Cu concentration varied significantly among the three sampling times in all groups (*p* < 0.001). In each group, the Cu values decreased considerably at the 24‐h sampling time following surgery, and the third sample showed a significant increase at the time of discharge compared to postoperative values. The Cu values at the time of discharge were higher than preoperative values, although not significant (Table 4). There was no significant difference among the three groups of patients regarding the induction of changes in Cu values (*p* = 0.434).

**TABLE 4:** Comparison of serum Cu and Zn values (mean ± SD) before and after surgery, and at the time of discharge in the three groups of patients

Group	Sampling time	Copper (μg/dl)	Zinc (μg/dl)	Zn/cu
On‐pump	Preoperative	116.35 ± 24.81^a^	73.65 ± 26.85^a^	0.63 ± 0.14^a^
	24 h Postoperative	90.47 ± 19.94^b^	39 ± 15.19^b^	0.43 ± 0.14^b^
	At discharge	121.59 ± 30.08^a^	64.29 ± 24.54^a^	0.53 ± 0.16^ab^
Off‐pump	Preoperative	114.92 ± 28.45^a^	84.84 ± 18.43^a^	0.77 ± 0.19^a^
	24 h Postoperative	92.04 ± 24.31^b^	48.32 ± 13.04^b^	0.55 ± 0.15^b^
	At discharge	125.68 ± 21.75^a^	74.64 ± 17.24^c^	0.61 ± 0.17^b^
Valve	Preoperative	117.70 ± 39.18^a^	86.73 ± 20.02^a^	0.97 ± 1.25^a^
	24 h Postoperative	87.24 ± 22.77^b^	49.41 ± 10.39^b^	0.61 ± 0.20^a^
	At discharge	132.08 ± 30.82^a^	78.03 ± 18.63^c^	0.61 ± 0.13^a^

*Note*: In a column for each group, values not sharing a common letter differ significantly according to the Bonferroni multiple comparison tests at *p* < 0.05.

### 3.4 Zn values

There was no significant difference in baseline Zn values for the off‐ and on‐pump CABG and valve replacement groups of patients (*p* = 0.361). Analysis by repeated measure ANOVA showed that Zn concentration varied significantly among the three sampling times in all groups (*p* < 0.001). In each group, the Zn values decreased significantly at the 24‐h sampling time following surgery, and the third sample showed a significant increase at the time of discharge compared to postoperative values. In the off‐pump CABG and valve replacement groups, zinc values remained significantly lower at discharge time than preoperative values (Table 4).

There was no significant difference among the three groups of patients regarding the induction of changes in zinc values (*p* = 0.986). The main effect of the grouping factor was significant (*p* = 0.004), and multiple comparisons using the Scheffe test showed that zinc value in the on‐pump group was significantly lower than those off‐pump CABG (*p* = 0.038) and valve replacement groups (*p* = 0.005).

### 3.5 Zn/cu ratio

There was no significant difference in baseline Zn/Cu ratio for the off‐ and on‐pump CABG and valve replacement groups of patients (*p* = 0.068). Analysis by a repeated measures test showed that the changes in Zn/Cu ratio were significantly different among the three samples in each group (*p* = 0.006). In each group, the Zn/Cu ratio decreased at the 24‐h sampling time following surgery, and the third sample showed an increase at the time of discharge compared to postoperative values, although remaining lower than preoperative ratios. There was no significant difference among on‐ and off‐pump CABG and valve replacement groups regarding the induction of changes in Zn/Cu ratios (*p* = 0.683) (Table 4).

There was no correlation between CPB time or aortic clamp time and Zn and Cu level changes before and after valve replacement surgery (Table 5). This was also true in the on‐pump CABG, except that there was a significant positive correlation between Cu changes and the duration of CPB (*p* < 0.05; *r* = 0.331) (Table 6). Longer durations of CPB were associated with greater decreases in the Cu level after On‐pump CABG. There was also no correlation between CPB time or aortic clamp time and Zn and Cu level changes before surgery and at the time of discharge.

**TABLE 5:** Correlation between CPB time or aortic clamp time and zinc and copper level changes before and after valve replacement surgery

Variable	Zinc changes		Copper changes	
	*r*‐value	*p*‐value	*r*‐value	*p*‐value
CPB time (min)	0.127	0.422	0.072	0.650
Aortic clamp time (min)	0.162	0.304	0.148	0.349

**TABLE 6:** Correlation between CPB time or aortic clamp time and zinc and copper level changes before and after on‐pump CABG surgery

Variable	Zinc changes		Copper changes	
	*r*‐value	*p*‐value	*r*‐value	*p*‐value
CPB time (min)	0.265	0.086	0.331	0.030
Aortic clamp time (min)	0.121	0.463	0.173	0.292

The changes in levels of Cu and Zn preoperatively and at the time of discharge were compared in terms of hypertension, smoking, diabetes, and dyslipidemia. The results showed no significant difference in mean Cu and Zn changes in terms of the above factors in each surgery group. Except that in the off‐pump group, mean changes of Zn were higher in patients without a history of hypertension compared to hypertensive patients (17.16 ± 14.83 vs. 3.7 ± 16.56 μg/dl; *p* = 0.045). Also, in the off‐pump group, patients with a history of diabetes had higher mean Zn changes than non‐diabetic patients (23.12 ± 16.3 vs. 4.11 ± 13.71 μg/dl; *p* = 0.006).

## 4 DISCUSSION

This study aimed to describe changes in Zn and Cu levels in patients undergoing off‐ and on‐pump CABG and valve replacement surgery, which is known to enhance the production and release of oxidants. Trace elements, including selenium, Zn, and Cu, may be protective against cardiovascular disease (Cuzzocrea et al., 2001). In our study, Zn concentration varied significantly among the three sampling times in all groups, and serum levels of Zn decreased considerably during the 24‐h sampling time following surgery. These results were in accordance with other studies, observing that the serum Zn levels after CABG with CPB decreased significantly (Al‐Bader et al., 1998; Fraser et al., 1989; Gormus et al., 2005; Yan et al., 2013). Kurian et al. (2008) also observed decreased Zn levels during the ischemic stage of CABG surgery. In the early revascularization period, the concentrations of Zn in the serum were elevated, and they were observed to be decreased once more in the late revascularization stage (Kurian & Paddikkala, 2007). This depletion in serum Zn might have resulted in the depletion of essential enzymes, which protect cell membranes from damage by free radicals. Other studies on patients undergoing valve replacement surgery also reported decreased postoperative concentrations of Zn (Nuutinen et al., 1981; Yan et al., 2013). More studies on the depth of Cu and Zn variations in patients undergoing surgical aortic valve replacement have indicated that Cu increased by 45% and a 10‐fold increase in the concentrations of Zn in the sclerotic compared with the control valves (Nyström‐Rosander et al., 2002).

Although the reduction in Zn values can be ascribed to a direct impact of hemodilution elicited by CPB (Caputo et al., 1998), in the present study, this reduction was also observed in the off‐pump CABG group. The decrease in serum Zn may also be related to a redistribution of Zn to the site of tissue injury (King, 2011). It has been shown that radiolabeled Zn localizes in actively healing wounds, reaching maximal concentrations in tissue within 24 to 48 h after injury (Savlov et al., 1962). Zinc can also decrease serum as a nonspecific reaction to stress (Mertens et al., 2015). In addition, surgical trauma increases corticosteroids, decreasing serum Zn concentration (Pacilli & Willetts, 2015).

In the current study, serum Zn values at the time of discharge (mean 8 days post‐operation) showed a significant increase compared to postoperative values, although remaining lower than preoperative values. Likewise, in a study conducted by Al Bader et al., Zn levels remained low throughout the study period (5th postoperative day) (Al‐Bader et al., 1998). Antila et al. (1990). also observed significantly lower Zn levels in CABG patients 2 months after the operation (Antila et al., 1990). This suggests that a supplementary Zn administration could be appropriate during open‐heart surgeries.

The changes in Cu values were also significantly different among the three sampling times. In each group, the preoperative Cu values decreased considerably at the 24‐h sampling time following surgery. This immediate postoperative decrease of Cu in CABG patients was parallel to other studies (Antila et al., 1990; Fraser et al., 1989; Gormus et al., 2005; Jeremy et al., 2002; Yan et al., 2013). Kurian et al. (2008) observed increased Cu levels during the ischemic stage of CABG surgery and in the early revascularization period, although afterward, Cu levels were observed to be decreased in the late revascularization stage (Kurian et al., 2008). Other studies on patients undergoing valve replacement surgery also reported reduced postoperative concentrations of Cu (Nuutinen et al., 1981; Yan et al., 2013). The changes in serum Cu may be attributable to hemodilution or responses to surgical trauma. Copper concentrations in serum increased at the time of discharge (mean 8 days post‐operation), with no significant differences between the surgery groups, and this increase, which has been quoted as an acute‐phase component (Myers et al., 1984), may be primarily related to increased synthesis of ceruloplasmin (Fraser et al., 1989). Therefore, it is possible that serum Cu levels decrease immediately after surgery, as a result of the combined effect of hemodilution and increased consumption in response to ischemia–reperfusion and oxidative stress; then increase to supra‐normal levels several days after surgery as a consequence of inflammation, as observed by other researchers (Fraser et al., 1989; Jeremy et al., 2002; Nuutinen et al., 1981) and similar to the present study.

The present study showed no significant difference among on‐ and off‐pump CABG and valve replacement groups regarding the induction of changes in Zn and Cu values. However, the results showed that the Zn concentration in the on‐pump group was significantly lower than in the off‐pump CABG and valve replacement counterparts.

Recent attention has focused on the effect of the off‐pump CABG technique working on the beating heart without extracorporeal circulation (Gasz et al., 2004; Raja & Dreyfus, 2008; Wan et al., 1999). Comparative studies have proved that off‐pump CABG surgery may reduce mortality and morbidity (Afilalo et al., 2012; Al‐Ruzzeh et al., 2003). According to these studies, the off‐pump CABG operation is associated with notable decreases in postoperative blood loss and need for transfusion and with a shortened time period on ventilatory support and in intensive care. Concerning our data in the present study (Table 4), decreased Zn trends were observed in each group; however, it was more remarkable in the on‐pump CABG group. The mechanisms explaining these observations may be related to the several events occurring during CPB, which are material dependent (exposure of blood to non‐physiologic surfaces) or material independent (surgical trauma, ischemia–reperfusion, and changes in the body temperature) (Falsoleiman et al., 2011).

Our study also has some limitations. First, we performed this study on relatively small samples of patients which were not fully matched for gender, age, or clinical history. Thus, more studies with larger sample sizes will be necessary to confirm our results. In addition, we did not measure the levels of oxidative stress or inflammatory markers in our study after CABG or valve replacement surgery. Evaluation of these markers would be expected to give better insight into the effects of Zn and Cu after cardiac surgeries.

## 5 CONCLUSION

Our data indicate that Cu and Zn concentrations are markedly decreased 24 h after CABG, and valve replacement surgery and thereafter rose at the discharge time. Levels of Cu and Zn were not significantly different for patients undergoing surgery with or without CPB. This may suggest that a supplementary Zn and Cu administration could be beneficial during open‐heart surgeries. However, more long‐term studies with more patients are needed to confirm this hypothesis.

## AUTHOR CONTRIBUTIONS

Zahra Moravvej: Investigation, Data Curation, Writing—Original Draft; Vafa Baradaran Rahimi: Writing—Original Draft, Writing—review & editing, Formal Analysis; Ali Azari: Conceptualization, Methodology, Investigation; Amir Ali Rahsepar: Methodology, Investigation; Majid Ghayour‐Mobarhan: Methodology, Investigation; Maryam Salehi: Formal Analysis, Data Curation; Leila Bigdelu: Conceptualization, Methodology, Funding Acquisition, Investigation, Supervision.

## FUNDING INFORMATION

This study was financially supported by the research council of the Mashhad University of Medical Sciences (Grant Number: 920908).

## CONFLICT OF INTEREST

The authors declare no conflict of interest.

## ETHICS STATEMENT

The study protocol was approved by the ethics committee of Mashhad University of Medical Sciences, and written informed consent was obtained from each participant.
